# Synthesis, X-Ray Structure, and Characterization of a Complex Containing the Hexakis(urea)cobalt(II) Cation and Lattice Urea Molecules

**DOI:** 10.1155/2007/51567

**Published:** 2008-01-02

**Authors:** Labrini Drakopoulou, Constantina Papatriantafyllopoulou, Aris Terzis, Spyros P. Perlepes, Evy Manessi-Zoupa, Giannis S. Papaefstathiou

**Affiliations:** ^1^Department of Chemistry, University of Patras, 265 04 Patras, Greece; ^2^Institute of Materials Science, National Centre of Scientific Research “Demokritos”, 153 10 Agia Paraskevi Attikis, Greece; ^3^Laboratory of Inorganic Chemistry, Department of Chemistry, National and Kapodistrian University of Athens, Panepistimiopolis,157 71 Zografou, Greece

## Abstract

The 12: 1 reaction of urea (U) with CoI_2_ in EtOH yielded the “clathrate-coordination” compound $[{\text{CoU}}_{6}]{\text{I}}_{2}\cdot$4U (**1**). The complex crystallizes in the monoclinic space group P2_1_/c. The lattice constants are *a* = 9.844(4), *b* = 7.268(3), *c* = 24.12(1) Å, and $\beta =98.12{\left(1\right)}^{\circ }$. The crystal structure determination demonstrates the existence of octahedral ${\left[{\text{CoU}}_{6}\right]}^{2+}$ cations, ${\text{I}}^{-}$ counterions, and two different types (two ${\text{U}}_{\text{1}}$ and two ${\text{U}}_{\text{2}}$) of hydrogen-bonded, lattice urea molecules. The ${\left[{\text{CoU}}_{6}\right]}^{2+}$ cations and the ${\text{U}}_{\text{1}}$ lattice molecules form two-dimensional hydrogen-bonded layers which are parallel to the *ab* plane. The ${\text{I}}^{-}$ anions are placed above and below each layer, and are hydrogen bonded both to ${\text{U}}_{\text{1}}$ molecules and ${\left[{\text{CoU}}_{6}\right]}^{2+}$ cations. Each ${\text{U}}_{\text{2}}$ molecule is connected to a ${\left[{\text{CoU}}_{6}\right]}^{2+}$ cation through an N–H⋯O hydrogen bond resulting in a three-dimensional network. Room temperature magnetic susceptibility and spectroscopic (solid-state UV/Vis, IR, Raman) data of **1** are discussed in terms of the nature of bonding and the known structure.

## 1. INTRODUCTION

**Scheme 1:**
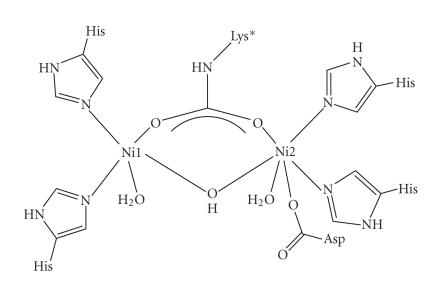
Schematic representation of the active site of urease.

**Scheme 2:**
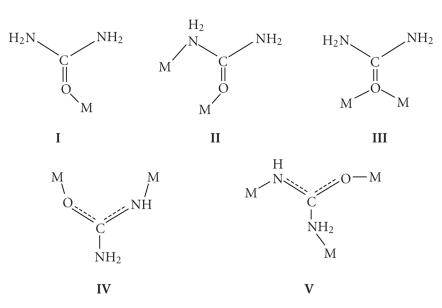
The crystallographically established coordination modes of urea (U) and its monoanion (H_2_NCONH^−^).

Urea, H_2_NCONH_2_ (hereafter abbreviated as U), is a very old compound, first isolated by Rouelle in 1773 and subsequently synthesized from inorganic materials by Wöhler in 1828. There is a renewed interest in the coordination chemistry of U and its substituted derivatives. These efforts are driven by a number of considerations, including the solution of pure chemical [1] and spectroscopic [2] problems, the desire to provide useful bioinorganic models for the intermediates in the catalytic mechanism of the metalloenzyme urease (for its active site see Scheme 1) [3, 4], and the goal to isolate functional complexes with interesting supramolecular structures [5]. Single-crystal X-ray crystallography has revealed [6] that U normally coordinates as a monodentate ligand through the oxygen atom (**I** in Scheme 2). In a very limited number of cases [7, 8], U behaves as an N,O-bidentate bridging ligand (**II** in Scheme 2), while in [Hg_2_Cl_4_U_2_] each U molecule bridges the two Hg^II^ atoms through only the oxygen atom [9] (**III** in Scheme 2). Of particular chemical/biological interest is the ability of U to undergo metal-promoted deprotonation [3, 10]; the monoanionic ligand H_2_NCONH^−^ adopts the $\mu_{2}$ (**IV** in Scheme 2) and $\mu_{3}$ (**V** in Scheme 2) coordination modes.

Free ureas have been among the central players in organic crystal engineering [11]. In contrast, little is known about the supramolecular architectures created by hydrogen bonding interactions between simple metal-urea complexes. By reacting metal ions with ureas that contain both an efficient coordination site and two hydrogen bonding functionalities, assembly can be dictated by intermolecular/interionic hydrogen bonding interactions. We have relatively recently [12–14] embarked on a program which has as a short-term goal the creation of supramolecular structures based on hydrogen bonding interactions between *simple* metal complexes with U or substituted ureas as ligands. Literature [5] reveals the existence of single-crystal X-ray and neutron structures for the impressive complex [CoU_6_]Br_2_ ⋅4U, which has been described as a “clathrate-coordination” compound. We were interested in investigating whether a similar iodide complex could exist; the present paper provides the answer to this question. Another goal of this work has been the study of the vibrational spectra of metal ion-U complexes, especially in view of the reassignment of the vibrational spectrum of free U [15].

## 2. EXPERIMENTS

All manipulations were performed under aerobic conditions using materials and solvents as received. IR spectra were recorded on a Perkin-Elmer PC16 FT-IR spectrometer with samples prepared as KBr pellets. Far-IR spectra were recorded on a Bruker IFS 113v FT spectrometer with samples prepared as polyethylene pellets. Solid-state (diffuse reflectance, DRS) electronic spectra in the 350–850 nm range were recorded on a Varian Cary 3 spectrometer equipped with an integration sphere. Room temperature magnetic susceptibility measurements were carried out by Faraday's method using a Cahn-Ventron RM-2 balance standardized with HgCo(NCS)_4_; diamagnetic corrections were estimated using Pascal's constants. C, H and N elemental analyses were performed with a Carlo Erba EA 108 analyzer, [CoU_6_]I_2_ ⋅4U (hereafter referred to as **1**).

To a stirred refluxing colorless solution of U (0.72 g, 12 mmol) in EtOH (30 cm^3^) was added solid CoI_2_ (0.31 g, 1.0 mmol) in small portions. The obtained blue solution was refluxed for further 15 minutes. A noticeable color change to pink occurred after cooling down. The solution began to deposit X-ray quality, orange crystals of **1** after 24 hours. When precipitation was judged to be complete, the product was collected by filtration, washed with a little cold EtOH (1-2 cm^3^) and Et_2_O $\left(2\times 5\;{\text{cm}}^{3}\right)$, and dried in air. The yield was 77% (based on the metal); found %: C, 13.22; H, 4.50; N, 30.48. Calc % for C_10_H_40_N_20_O_10_CoI_2_: C, 13.15; H, 4.42; N, 30.68; selected IR data (KBr, cm^−1^): 3450 (sh), 3438 (s), 3346 (m), 3438 (sh), 1685 (sh), 1666 (s), 1648 (sh), 1622 (m), 1578 (m), 1478 (m), 1444 (m), 1156 (m), 1050 (w), 780 (m), 617 (sh), 600 (w), 570 (m), 532 (m), 366 (m).

### 2.1. X-ray crystallography

X-ray data were collected at 298 K using a Crystal Logic Dual Goniometer diffractometer with graphite-monochromated Mo-*K_a_* radiation (λ=0.71073⁢ Å). Lorentz, polarization, and Ψ-scan absorption corrections were applied using Crystal Logic software. Symmetry equivalent data were averaged with $R_{\text{int}}=0.0202$, to give 3006 independent reflections from a total 3086 collected. The structure was solved by direct methods and refined by full-matrix least-squares on F^2^, using 3006 reflections and refining 276 parameters. All nonhydrogen atoms were refined anisotropically. All hydrogen atoms bonded to nitrogen atoms were located by difference maps and their positions were refined isotropically. There were no significant residual peaks in the electron density map. Details of the data collection and refinement are given in Table 1. Topological analysis of the nets was performed using TOPOS program package [16, 17].

## 3. RESULTS AND DISCUSSION

### 3.1. Synthetic comments

The CoI_2_/U reaction system was synthetically investigated in the past. Depending on the reaction and crystallization conditions, the complexes [CoU_6_](I_8_) [18], [CoU_6_](I_3_)_2_ ⋅2U [19], [CoU_2_(H_2_O)_4_][CoI_4_]⋅H_2_O [20] and [CoU_4_(H_2_O)_2_]I_2_ [20] were isolated and structurally characterized. In these four complexes, the U : Co^II^ ratio varies from 1 : 1 to 8 : 1. We wondered if complexes with a higher U to Co^II^ ratio, that is, a higher urea percentage, would be capable of existence. Thus, the 12 : 1 reaction of U and CoI_2_ in refluxing EtOH gave orange crystals of compound [CoU_6_]I_2_ ⋅4U (**1**) in very good yield (ca. 80%). The reaction can be represented by the stoichiometric equation (1):

(1) $${\text{CoI}}_{2}+10\;\text{U}\overset{\text{EtOH}}{\underset{\text{T}}{\to }}\underset{1}{\left[{\text{CoU}}_{6}\right]}{\text{I}}_{2}\cdot 4\text{U.}$$

The “wrong” stoichiometry employed, that is, U : CoI_2_ = 12 : 1 instead of 10 : 1 (required by (1)), is necessary for the precipitation of pure **1**. The 10 : 1 reaction ratio in EtOH under reflux leads to a mixture of **1** and [CoU_6_](I_3_)_2_ ⋅2U [19)]; the identity of the latter was confirmed by unit cell determination. The same complex cannot be prepared in other solvents; use of MeCN leads to complexes [CoU_2_(H_2_O)_4_][CoI_4_] [20] and [CoU_4_(H_2_O)_2_]I_2_ [20] mentioned above, and to a third product (analyzed as [CoU_6_]I_2_) which has yet to be structurally characterized.

### 3.2. Description of structure

**Figure 1:**
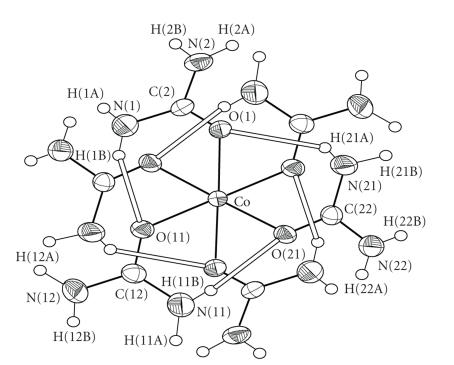
An ORTEP representation of the cation [CoU_6_]^2+^ present in complex **1**. Open bonds indicate intramolecular hydrogen bonds. The symmetry-equivalent atoms are not labeled.

The structure of **1** consists of octahedral [CoU_6_]^2+^ cations, I^−^ anions and lattice urea molecules (four lattice urea molecules per cation). The structure of the [CoU_6_]^2+^ cation is shown in Figure 1, and selected bond lengths and angles are listed in Table 2. The Co^II^ ion sits on an inversion centre and is surrounded by six O-bonded urea ligands. The octahedral coordination around the Co^II^ atom is slightly distorted, as evidenced by the Co–O bond distances and O–Co–O bond angles. The Co–O bond distances in **1** are comparable to those in other [CoU_6_]^2+^ complexes [18–20]. The urea ligands in **1** are coordinated in a bent fashion, with the C–O–Co angles ranging from 130.3(2)° to 133.6(2)°. This is the usual way of coordination of urea and its derivatives [2, 12]. There are six strong intramolecular (intracationic) hydrogen bonds with atoms N(1), N(11), and N(21) (and their symmetry equivalents) as donors, and atoms O(1), O(11), and O(21) (and their symmetry equivalents) as acceptors. These six intramolecular hydrogen bonds give a great thermodynamic stability which is responsible for the formation of [CoU_6_]^2+^.

**Figure 2:**
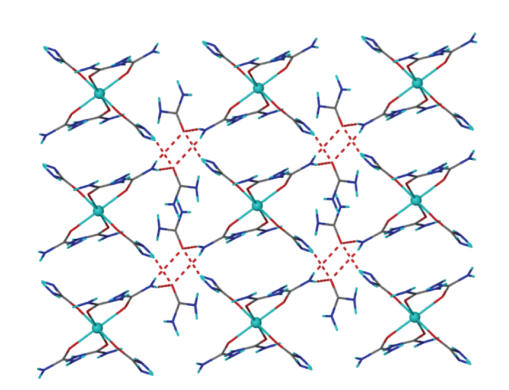
A view of the 2D network formed by hydrogen bonding between the [CoU_6_]^2+^ cations and the U_1_ molecules in **1**. Only the intermolecular hydrogen bonds are shown.

**Figure 3:**
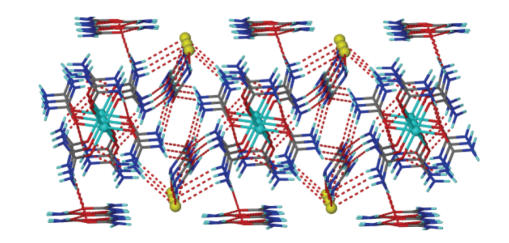
A view of the 3D network formed by hydrogen bonding between the “[CoU_6_]I_2_·2U_1_” layers and the U_2_ tapes in **1**.

We have up to now discussed aspects of the molecular structure of **1**. Figures 2 and 3 provide views of the hydrogen-bonded network of [CoU_6_]I_2_ ⋅4U. Metric parameters for the intermolecular hydrogen bonds present in the crystal structure of **1** have been included in Table 3. The asymmetric unit of **1** contains five crystallographically independent urea molecules; three of them are coordinated to the Co^II^ atom while the other two (hereafter termed U_1_ and U_2_) are lattice molecules. The [CoU_6_]^2+^ cations and the U_1_ lattice molecules form two-dimensional (2D) by about 12 Å along the *c* axis (Figure 2). Each [CoU_6_]^2+^ cation is hydrogen bonded to six U_1_ molecules through the hydrogen-bonded layers which are parallel to the *ab* plane and separated N(1)–H(1A)⋯O(31)b (b 1−*x*, 0.5 + *y*, 0.5−*z*), N(22)–H(22A)⋯O(31)d (d *x*, 0.5−*y*, −0.5 + *z*) and N(22)–H(22B)⋯O(31)e (e −*x*, 0.5 + *y*, 0.5−*z*) (and their symmetry equivalents) hydrogen bonds, within a layer, with each U_1_ acting as hydrogen bond acceptor through the O(31) atom and connecting three different [CoU_6_]^2+^ cations. The I^−^ anions are placed above and below each layer and are hydrogen bonded both to U_1_ molecules and [CoU_6_]^2+^ cations. Each I^−^ accepts five hydrogen bonds connecting two [CoU_6_]^2+^ cations through the N(2)–H(2B)⋯Ic (c *x*, *y*, −1 + *z* and N(21)–H(21B)⋯Ih (h −1 + *x*, *y*, −1 + *z*) hydrogen bonds and two U_1_ molecules through the N(31)–H(31A)⋯If (f 1−*x*, 0.5−*y*, 1.5−*z*), N(31)–H(31B)⋯Ig (g 1−*x*, 0.5 + *y*, 1.5−*z*) and N(32)–H(32A)⋯Ig hydrogen bonds. In this arrangement, each [CoU_6_]^2+^ is hydrogen bonded to four I^−^ anions while each U_1_ molecule to two I^−^ anions.

The U_2_ molecules are hydrogen bonded to each other through the N(41)–H(41A)⋯O(41)f and N(42)–H(42A)⋯O(41)g hydrogen bonds to form one dimensional tapes that run parallel to the *b* axis. The U_2_ tapes are parallel to the “[CoU_6_]I_2_ ⋅2U_1_” layers and the *ab* plane, and are separated by 9.844 Å along the *a* axis. Each U_2_ molecule is connected to a [CoU_6_]^2+^ cation through the N(2)–H(2A)⋯O(41)c hydrogen bond (Figure 3) resulting in a three-dimensional (3D) hydrogen-bonded network; the U_2_ tapes are placed within the “[CoU_6_]I_2_ ⋅2U_1_” layers and connect them to the third dimension.

**Figure 4:**
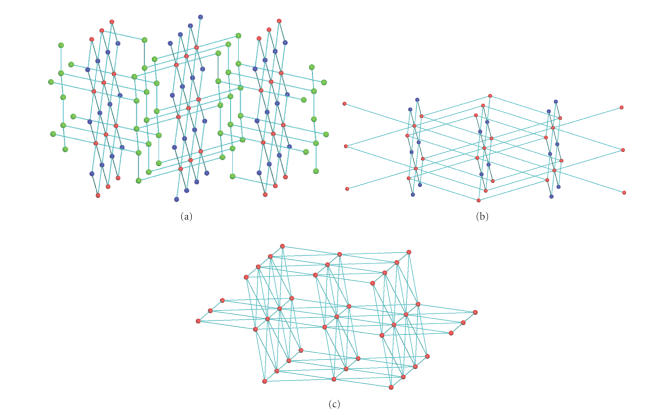
Views of (a) the trinodal 3D network, (b) the simplified binodal network, and (c) the simplified uninodal 12-connected network of **1**.

From the topological point of view, the [CoU_6_]^2+^ cations and the U_1_ molecules form a 2D framework, with a (4,4)-topology and two different types of 4-connected nodes. Each [CoU_6_]^2+^ serves as a 4-connected node within the 2D net where the other type of 4-connected node is situated on the centre of the ${\text{R}}_{\text{4}}^{\text{2}}$(8) ring formed by two [CoU_6_]^2+^ and two U_1_ molecules (Figure 2). The “[CoU_6_]^2+^ ⋅2U_1_” layers are connected to the third dimension only through the [CoU_6_]^2+^ cations, which therefore serve as 6-connected nodes within the 3D framework. Each U_2_ molecule is hydrogen bonded to two other U_2_ molecules and at the same time to one [CoU_6_]^2+^. In this arrangement, each U_2_ molecule serves as a 3-connected node within the 3D framework. Therefore, the 3D framework is a trinodal net with 3-, 4-, and 6-connected nodes and a unique (6^3^)_2_ $\left(4^{4}\cdot 6^{2}\right)$ $\left(4^{4}\cdot 6^{10}\cdot 8\right)$ topology (Figure 4(a)). If we consider that the U_2_ molecules simply connect the 2D layers and merge them to the [CoU_6_]^2+^ nodes, then we can simplify the 3D framework to a binodal net with 4- and 8-connected nodes and a unique $\left(4^{4}\cdot 6^{2}\right)$ $\left(4^{16}\cdot 6^{12}\right)$ topology (Figure 4(b)). The latter network can be simplified further to a uninodal net by merging the 4-connected nodes to the 8-connected nodes resulting in a 12-connected network with a unique $3^{18}\cdot 4^{44}\cdot 5^{4}$ topology (Figure 4(c)). Interestingly, the new 12-connected network has the same coordination sequence with the **fcu** net [21].

Complex **1** joins a handful of structurally characterized complexes containing the octahedral cation [CoU_6_]^2+^ [5, 18, 19, 22]; it is isostructural with [CoU_6_]Br_2_ ⋅4U [5] and [NiU_6_]I_2_ ⋅4U [23].

### 3.3. Physical and spectroscopic characterization

The room temperature value of the effective magnetic moment $\left(\mu_{\text{eff}}\right)$ for **1** is 4.93 BM per metal ion, to be compared with the spin-only $(g=2)\;\mu_{\text{eff}}$ value of 3.87 BM. This value is within the range observed for six-coordinate, high-spin cobalt(II) complexes [24]. Because of the intrinsic orbital angular momentum in the octahedral ground state T41g(F), there is consistently a considerable orbital contribution and $\mu_{\text{eff}}$ values for such compounds around room temperature are between 4.7 and 5.2 BM.

The solid-state electronic spectral data of **1** also indicate an octahedral stereochemistry around cobalt(II). A multiple structured bond, assigned to T41g(F)→T41g(P), is seen in the visible region near 530 nm with a clear shoulder at 474 nm [25]. The multiple structure arises from the admixture of spin forbidden transitions to doublet states mainly derived from G2 and H2. The T41g(F)→A42g transition appears as an ill-defined shoulder at ~665 nm [25]. The calculated ligand field parameters from the two transitions in the visible region are 10Dq = 8050 cm^−1^ and B = 865 cm^−1^ [25]; these values are typical for a Co^II^O_6_ chromophore.

**Scheme 3:**
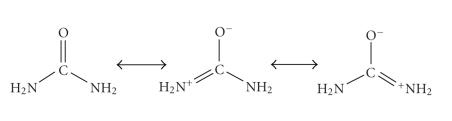
Resonance forms of urea.

The full vibrational analysis of crystalline U has been published [15]. Table 4 gives diagnostic IR bands of the free ligand and **1**. Assignments have been given in comparison with the data obtained for the free, that is, uncoordinated, U [15] and its manganese(II) complexes [2]. The bands with *ν*(CN) character are situated at higher wavenumbers in the spectrum of **1** than for free U, whereas the *ν*(CO) band shows a frequency decrease. These shifts are consistent with oxygen coordination, suggesting the presence of ^+^N=C–O^−^ resonant forms [15], see Scheme 3. Upon coordination *via* oxygen, the positively charged metal ion stabilizes the negative charge on the oxygen atom; the NCO group now occurs in its polar resonance form and the double bond character of the CN bond increases, while the double bond character of the CO bond decreases, resulting in an increase of the CN stretching frequency with a simultaneous decrease in the CO stretching frequency [2, 12–14]. The appearance of two bands for each of the $\delta_{\text{as}}\left(\text{N}{\text{H}}_{2}\right)$ and $\delta_{\text{s}}\left(\text{N}{\text{H}}_{2}\right)$ modes in **1** may indicate the existence of two types of U molecules, coordinated and uncoordinated (lattice). However, the appearance of one band for each of the other modes suggests that the coordinated and lattice (but hydrogen bonded) U molecules of **1** cannot, in general, be differentiated in the vibrational spectrum. This is not unexpected, bearing in mind that the hydrogen bonds have an effect similar to that of coordination on the shifts of the CO and CN stretching vibrations. This can be explained [15] by regarding the hydrogen bond as a donor-acceptor “complex,” with the oxygen atom as the donor and the hydrogen atom as the acceptor. The appearance of one IR-active *ν*(CoO) vibration at 366 cm^−1^ (*F*_1*u*_ under *O_h_*) in the low-frequency region of **1** reflects the *trans* octahedral stereochemistry of [CoU_6_]^2+^ [13, 26].

## 4. CONCLUSIONS

This work has shown that the hexakis(urea)cobalt(II) cation can act as a hydrogen bonding building block with multi-fold connectivity linking I^−^ anions and U molecules to generate a 3D architecture. We are presently pursuing our prediction that this cation will form hydrogen bonding contacts to a variety of inorganic and organic anions to generate a rich diversity of networks. Complex **1**, which is isostructural with its bromide analogue [5], is becoming the fifth structurally characterized member of the Co^II^/I^−^/U family of complexes [18–20], emphasizing the rich molecular and supramolecular chemistry of this system.

The role of metal ions in supramolecular systems may simply be to act as coordination centers providing a template for the formation of a rigid framework of remote hydrogen bonding sites. Alternatively, the metal ion may exert an electronic effect on the individual proton and acceptor sites, and influence hydrogen bonding in a more subtle manner. The latter effect lets us believe that the reactions of other metal ions with urea may lead to the formation of complexes with novel supramolecular structures.

## Figures and Tables

**Table 1:** Crystal data and structure refinement for **1**.

Empirical formula	C_10_H_40_CoI_2_N_20_O_10_
Formula weight	913.35
Crystal size	0.10×0.20×0.50
Crystal system	Monoclinic
Space group	P2_1_/c
θ range for data collection °	1.71≤θ≤24.97
*a*, Å	9.844(4)
*b*, Å	7.268(3)
*c*, Å	24.12(1)
α, °	90
β, °	98.12(1)
γ, °	90
*V*, Å^3^	1708(1)
*Z*	2
$\rho_{\text{calcd}}$, g cm^−3^	1.775
μ, mm^−1^	2.380
*GOF*	1.057
*R*1^a^	0.027
*wR*2	0.066

^a^ *I*> 2*σ*(*I*).

**Table 2:** Selected dond lengths (Å) and angles (°) for **1**; symmetry transformation used to generate equivalent atoms: a 1−*x*, −*y*, −*z*; atoms C(32), O(31), N(31), N(32) and C(42), O(41), N(41), N(42) belong to the two crystallographically independent lattice U molecules (U_1_ and U_2_, resp.).

Co–O(1)	2.092(2)	O(1)–Co–O(21)a	87.3(1)
Co–O(11)	2.091(2)	O(11)–Co–O(21)	92.5(1)
Co–O(21)	2.110(2)	O(11)a–Co–O(21)	87.5(1)
O(1)–C(2)	1.246(4)	O(1)–Co–O(21)	92.7(1)
O(11)–C(12)	1.250(4)	O(1)a–Co–O(21)	87.3(1)
O(21)–C(22)	1.254(4)	O(21)a–Co–O(21)	180.0
C(2)–N(1)	1.323(5)	C(2)–O(1)–Co	133.6(2)
C(2)–N(2)	1.350(5)	C(12)–O(11)–Co	131.1(2)
C(12)–N(11)	1.325(6)	C(22)–O(21)–Co	130.3(2)
C(12)–N(12)	1.330(6)	O(1)–C(2)–N(1)	122.9(3)
C(22)–N(21)	1.321(6)	O(1)–C(2)–N(2)	119.9(4)
C(22)–N(22)	1.333(5)	N(1)–C(2)–N(2)	117.1(4)
O(31)–C(32)	1.234(4)	O(11)–C(12)–N(11)	122.8(4)
C(32)–N(32)	1.332(5)	O(11)–C(12)–N(12)	119.5(4)
C(32)–N(31)	1.346(5)	N(11)–C(12)–N(12)	117.7(4)
O(41)–C(42)	1.241(4)	O(21)–C(22)–N(21)	122.9(4)
C(42)–N(42)	1.332(5)	O(21)–C(22)–N(22)	120.1(4)
C(42)–N(41)	1.338(5)	N(21)–C(22)–N(22)	117.0(4)
O(11)–Co–O(11)a	180.0	O(31)–C(32)–N(32)	121.7(4)
O(11)–Co–O(1)	94.1(1)	O(31)–C(32)–N(31)	122.4(4)
O(11)a–Co–O(1)	85.9(1)	N(32)–C(32)–N(31)	115.8(4)
O(11)–Co–O(1)a	85.9(1)	O(41)–C(42)–N(42)	121.7(4)
O(1)–Co–O(1)a	180.0	O(41)–C(42)–N(41)	120.7(4)
O(11)–Co–O(21)a	87.5(1)	N(42)–C(42)–N(41)	117.5(4)

**Table 3:** Dimensions of the unique hydrogen bonds (distances in Å and angles in °) for complex **1**. ^†^

D^‡^–H⋯A^§^	D^‡^⋯A^§^	H⋯A^§^	<D^‡^HA^§^
N(1)–H(1B)⋯O(11)	2.879(1)	2.136(1)	153.26(4)
N(11)–H(11B)⋯O(21)	2.938(1)	2.228(1)	162.15(3)
N(21)–H(21A)⋯O(1)	2.886(1)	2.317(1)	143.23(4)
N(1)–H(1A)⋯O(31)b	2.955(1)	2.097(1)	166.58(1)
N(2)–H(2A)⋯O(41)c	3.076(1)	2.253(1)	149.41(3)
N(22)–H(22A)⋯O(31)d	2.982(1)	2.229(1)	170.70(6)
N(22)–H(22B)⋯O(31)e	3.075(1)	2.463(1)	133.71(2)
N(41)–H(41A)⋯O(41)f	3.001(1)	2.193(1)	171.64(1)
N(42)–H(42A)⋯O(41)g	2.938(1)	2.175(1)	164.66(1)
N(2)–H(2B)⋯Ic	3.665(1)	3.049(1)	146.31(2)
N(21)–H(21B)⋯Ih	3.616(1)	2.676(1)	172.38(6)
N(31)–H(31A)⋯If	3.853(1)	2.997(1)	172.59(1)
N(31)–H(31B)⋯Ig	3.871(1)	3.028(1)	159.58(6)
N(32)–H(31A)⋯Ig	3.822(1)	3.000(1)	151.85(6)

^†^Symmetry transformation used to generate equivalent atoms: b 1−*x*, 0.5 + *y*, 0.5−*z*; c *x*, *y*, −1 + *z*; d *x*, 0.5−*y*, −0.5 + *z*; e−*x*, 0.5 + *y*, 0.5−*z*; f 1−*x*, −0.5−*y*, 1.5−*z*; g 1−*x*, 0.5 + *y*, 1.5−*z*; h −1 + *x*, *y*, −1 + *z*.

^‡^D = donor atom.

^§^A = acceptor atom.

**Table 4:** Most characteristic and diagnostic IR fundamentals (cm^−1^) for U and complex **1**.

Assignment	U	**1**
$v_{\text{as}}$(NH_2_)	3450, 3444	3446, 3438
$v_{\text{s}}$(NH_2_)	3349, 3341	3346, 3335
$\delta_{\text{s}}$(NH_2_)	1683	1685, 1666
$\delta_{\text{as}}$(NH_2_)	1625	1648, 1622
v(CO)	1601	1578
$v_{\text{as}}$(CN)	1466	1478
$v_{\text{s}}$(CN)	1003	1018
